# Modeling the Impact of Tuberculosis Control Strategies in Highly Endemic Overcrowded Prisons

**DOI:** 10.1371/journal.pone.0002100

**Published:** 2008-05-07

**Authors:** Judith Legrand, Alexandra Sanchez, Francoise Le Pont, Luiz Camacho, Bernard Larouze

**Affiliations:** 1 U 707, INSERM, Paris, France; 2 UMR-S 707, Université Pierre et Marie Curie-Paris 6, Paris, France; 3 Department of Infectious Disease Epidemiology, Imperial College, London, United Kingdom; 4 Coordenação de Gestão em Saúde, Secretaria de Estado de Administração Penitenciária de Rio de Janeiro, Rio de Janeiro, Brasil; 5 Departamento de Epidemiologia e Metodos Quantitativos, Escola Nacional de Saude Publica, Fundação Oswaldo Cruz, Rio de Janeiro, Brasil; Instituto de Medicina Tropical Alexander Von Humboldt, Peru

## Abstract

**Background:**

Tuberculosis (TB) in prisons is a major health problem in countries of high and intermediate TB endemicity such as Brazil. For operational reasons, TB control strategies in prisons cannot be compared through population based intervention studies.

**Methodology/Principal Findings:**

A mathematical model is proposed to simulate the TB dynamics in prison and evaluate the potential impact on active TB prevalence of several intervention strategies. The TB dynamics with the ongoing program was simulated over a 10 year period in a Rio de Janeiro prison (TB prevalence 4.6 %). Then, a simulation of the DOTS strategy reaching the objective of 70 % of bacteriologically-positive cases detected and 85 % of detected cases cured was performed; this strategy reduced only to 2.8% the average predicted TB prevalence after 5 years. Adding TB detection at entry point to DOTS strategy had no major effect on the predicted active TB prevalence. But, adding further a yearly X-ray mass screening of inmates reduced the predicted active TB prevalence below 1%. Furthermore, according to this model, after applying this strategy during 2 years (three annual screenings), the TB burden would be reduced and the active TB prevalence could be kept at a low level by associating X-ray screening at entry point and DOTS.

**Conclusions/Significance:**

We have shown that X-ray mass screenings should be considered to control TB in highly endemic prison. Prisons with different levels of TB prevalence could be examined thanks to this model which provides a rational tool for public health deciders.

## Introduction

All over the world, tuberculosis (TB) is a public health problem in prisons due to the fact that many inmates come from communities at high risk of TB, to their living conditions in prisons and to the insufficiencies of prisons' health services [1]. This problem is particularly critical in countries of high and intermediate TB endemicity such as Brazil. In Rio de Janeiro (RJ) state prisons, the 2005 annual TB incidence rate was as high as 3 500/100 000 [2], 35 times higher than in the general population of the state [3]. Recent X-ray surveys performed in three RJ prisons found prevalence rates of active TB ranging from 4.6 to 8.6% [4], [5].

In addition to the universal World Health Organization (WHO) Directly Observed Treatment Short-course (DOTS) strategy [6], several measures have been proposed by WHO and the Red Cross to control TB in the prisons [7] including mass screening of prisoners based on symptoms [8] and the systematic detection of TB at entry point, commonly used in high income countries [9], [10]. However, the respective efficacy of these measures and of their combinations remains to be demonstrated, particularly in countries of high and intermediate TB endemicity [11]. But, in the context of prisons, comparative intervention studies to measure the efficacy of control strategies would be unfeasible and would raise ethical questions. Therefore, in order to explore the impact of several TB control strategies on the prevalence of active TB, we developed a mathematical model of TB dynamics in prisons. In the present study, parameter values were drawn from the medical literature and from epidemiological studies previously conducted in one of the RJ prison units [4].

## Materials and Methods

### Model

**Figure 1:**
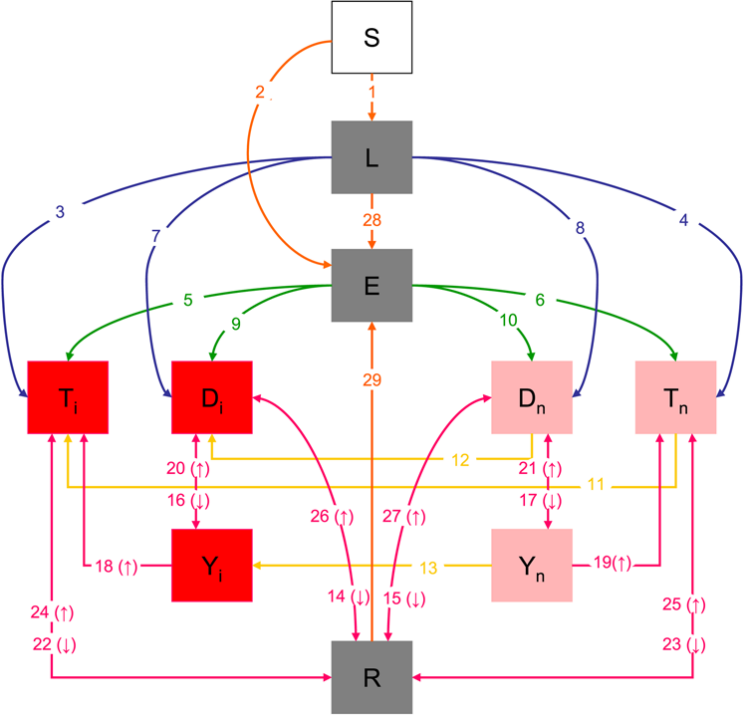
Structure of the mathematical model for the dynamics of tuberculosis in prison. Each box represents a compartment: Susceptible individuals (S), latent fast progressors (E), latent slow progressors (L), cured individuals (R), infectious/non-infectious cases who will be detected and treated (D_i_/D_n_), infectious/non-infectious cases who will not be detected and treated (T_i_/T_n_), infectious/non-infectious treated cases with treatment failure (Y_i_/Y_n_). Red boxes represent a disease-infectious state, pink boxes represent a disease-non infectious state and grey boxes represent infected individuals without disease. Entries and discharges in and out of the prison are not represented on this figure.

Based on previously published works [12]–[19], we developed a stochastic compartmental model where the population is distributed into 10 groups (see figure 1). This model allows the simulation of infection and re-infection, detection and treatment of cases, treatment failure, death from tuberculosis, self cure and incarceration of new prisoners. Susceptible individuals (S) can be infected by infectious TB cases (see figure 1, transitions 1–2). Infected cases can be either fast (E) or slow (L) progressors, the rate of progression from latency to active TB being greater for fast progressors (transitions 3–10). Active TB cases are divided into four groups according to whether they will be detected and treated thanks to passive detection (D) or not (T) and whether they are infectious (subscript i) or not (subscript n). However, non infectious cases can become infectious (transitions 11–13). After an average duration of 1/k years, cases in D states are detected and move to the recovered compartment (R) if the treatment is successful (transitions 14–15). Otherwise, they move to the treatment failure compartment (Y_i_ and Y_n_, transitions 16–17) before re-entering compartments T or D (transitions 18–21). Undetected TB cases (T_i_ and T_n_) can be self-cured (transitions 22–23). Recovered individuals (R) can relapse (transitions 24–27). Latent slow progressors (L) and cured individuals (R) can be re-infected by infectious cases (transitions 28–29). The rates of entries in compartments depend on the prevalence of TB infection and active TB at entry point and on the simulated TB control strategy. The rate of discharge in each compartment is proportional to the size of this compartment. Parameter definitions are given in table 1 and detailed transition rates are given in table 2. We considered that smear-positive and smear-negative/culture positive cases are infectious and that smear negative/culture negative TB cases are not.

**Table 1:** Definitions and values of model parameters

Parameter	Definition	Current scenario	Values in S1–S7	Distribution for LHS			Units	References
				min	peak†	max		
**N**	Number of inmates at the beginning of simulations	1000	1000				person	
**β**	Transmission rate	11.10^−3^	11.10^−3^	10.10^−3^		15.10^−3^	/person/year	[15], [17], [19], [20]
**p**	Proportion of fast progressors	0.14	0.14	0.08	0.14	0.25		[15], [21]
**m**	Partial immunity afforded by previous infection	0.41	0.41	0.4		0.9		[15], [21]
**τ_1_**	Rate at which slow progressors develop TB	0.00256	0.00256	0.00256		0.00527	/year	[12]
**τ_2_**	Rate at which fast progressors develop TB	0.9638	0.9638	0.76		0.99	/year	[21]
**θ**	Proportion of TB cases who become infectious	0.65	0.65	0.5	0.65	0.85		[12]
**ω**	Rate of smear conversion	0.015	0.015	0		0.02	/year	[15]
**g**	Proportion of treated cases who are cured	0.65	0.85					UD‡
**k**	Rate at which cases in D_i_ and D_n_ (see figure 1) are detected and treated	3.43	3.43	2.4		6.0	/year	PC§
**δ**	Rate of relapse	0.01	0.01	0	0.01	0.03	/year	[12]
**σ_1_**	Rate of self cure for non treated infectious cases	0.058	0.058	0.021	0.058	0.086	/year	[12]
**σ_2_**	Rate of self cure for non treated non infectious cases	σ_1_	σ_1_				/year	[12]
**ϕ**	Untreated TB death rate	0.14	0.14	0.058	0.139	0.461	/year	[12]
**f_1_**	Proportion of infectious cases detected	0.43	0.7					PC
**f_2_**	Proportion of non infectious cases detected	0.34	0					PC
**π**	Inmates turnover	1/3	1/3				/year	UD
	Detection at entry point	No	See table 3					
**Se**	Sensitivity of the detection of smear+symptomatic cases at entry point		0.21	0.2		0.4		[4], [22]

* LHS: Latin Hypercube Sampling

† The parameter distribution for the Latin Hypercube Sampling is triangular when «peak» is specified and is uniform otherwise

‡ UD: Unpublished data obtained in the prison we studied. The proportion of treated cases who are cured was obtained from the follow up records of TB cases. The inmates' turnover was based on the administrative records.

§ PC: Personal communication A.Sanchez. Parameter k corresponds to the inverse of the average duration of the time from progression to active TB to detection. We set this average duration at 3 months and a half (k = 3.43) with a range 2 months-5 months in the Latin Hypercube Sample.

**Table 2:** Description of the transition rates in the model

Transition number	Transition	Transition rate
1	(S-1; L+1)	(1-p)βS(T_i_+Y_i_+D_i_)
2	(S-1; E+1)	pβS(T_i_+Y_i_+D_i_)
3	(L-1; T_i_+1)	τ_1_θ(1-f_1_)L
4	(L-1; T_n_+1)	τ_1_(1-θ)(1-f_2_)L
5	(E-1; T_i_+1)	τ_2_θ(1-f_1_)E
6	(E-1; T_n_+1)	τ_2_(1-θ)(1-f_2_)E
7	(L-1; D_i_+1)	τ_1_θf_1_L
8	(L-1; D_n_+1)	τ_1_(1-θ)f_2_L
9	(E-1; D_i_+1)	τ_2_θf_1_E
10	(E-1; D_n_+1)	τ_2_(1-θ)f_2_E
11	(T_n_-1; T_i_+1)	wT_n_
12	(D_n_-1; D_i_+1)	wD_n_
13	(Y_n_-1; Y_i_+1)	wY_n_
14	(D_i_-1; R+1)	gkD_i_
15	(D_n_-1; R+1)	gkD_n_
16	(D_i_-1; Y_i_+1)	(1-g)kD_i_
17	(D_n_-1; Y_n_+1)	(1-g)kD_n_
18	(Y_i_-1; T_i_+1)	(1-f_1_)Y_i_
19	(Y_n_-1; T_n_+1)	(1-f_2_)Y_n_
20	(Y_i_-1; D_i_+1)	f_1_Y_i_
21	(Y_n_-1; D_n_+1)	f_2_Y_n_
22	(T_i_-1; R+1)	σ_1_T_i_
23	(T_n_-1; R+1)	σ_2_T_n_
24	(R-1; T_i_+1)	(1-f_1_)δθR
25	(R-1; T_n_+1)	(1-f_2_)δ(1-θ)R
26	(R-1; D_i_+1)	θδf_1_R
27	(R-1; D_n_+1)	(1-θ)δf_2_R
28	(L-1; E+1)	p(1-m)β(T_i_+Y_i_+D_i_)L
29	(R-1; E+1)	p(1-m)β(T_i_+Y_i_+D_i_)R
	(S+1)	(eS)Ω
	(S-1)	ΠS
	(L+1)	(eL)Ω
	(L-1)	ΠL
	(E+1)	(eE)Ω
	(E-1)	ΠE
	(T_i_+1)	(eTi)Ω
	(T_i_-1)	ΠT_i_+ϕT_i_
	(T_n_+1)	(eT_n_)Ω
	(T_n_-1)	ΠT_n_+ϕTn
	(D_i_+1)	(eD_i_)Ω
	(D_i_-1)	ΠD_i_
	(D_n_+1)	(eD_n_)Ω
	(D_n_-1)	ΠD_n_
	(Y_i_+1)	(eY_i_)Ω
	(Y_i_-1)	ΠY_i_
	(Y_n_+1)	(eY_n_)Ω
	(Y_n_-1)	ΠY_n_
	(R+1)	(eR)Ω
	(R-1)	ΠR

We denote Ω the annual number of inmates entering the prison and eX the proportion of prisoners who enter the X compartment. Other parameters definitions are given in table 1. Transition numbers correspond to the numbers on figure 1.

Simulations of the model were performed using the Gillespie's first reaction method [23]. A transition rate, λ_i_, depending only on the present state of the population, is allocated to each transition between two compartments. At each iteration of the algorithm, a time τ_i_ is drawn from an exponential distribution with parameter λ_i_ for each transition. The next transition µ is the transition that has the minimum time to occurrence (τ_µ_). Then, counts in each compartment are updated accordingly.

### Background and source of data

The 35 RJ State prisons hold nearly 23 000 inmates. In the present study, we investigated the TB dynamics in one of the RJ prisons (around 1000 inmates) where the prevalence of active TB is 4.6% [4]. As in most of RJ prisons, cells are overcrowded (median: 33 inmates/cell) and poorly ventilated. The inmates are sentenced for at least 8 years but, due to movements of inmates among prisons and to incarceration/freeing, the annual turnover of inmates is around one third. The TB control is based on the DOTS strategy which includes case detection through quality assured-bacteriology and standardized treatment with supervision and patient support [6]. There is no TB detection at entry in prison and no mass screening in the prison. Prevalence of active TB and prevalence of TB infection in prison and at entry point were inferred from surveys carried out in RJ prison units. The values of other parameters were obtained from the literature (see table 1).

### Simulation of TB control strategies

We first simulated the evolution of active TB prevalence in the prison over a 10 year period if the current TB control strategy remains unchanged. For simulating this current scenario, we considered that, among prisoners entering the prison, the prevalence of latent TB infection (defined as a positive tuberculin skin test after excluding active TB cases) is 47.0% and that the prevalence of active TB (evaluated through X-ray screening and bacteriological diagnostic tests) is 1.5% (unpublished data). Among inmates, we considered a prevalence of latent TB infection at 60.6% and a prevalence of active TB at 4.6% [4]. Furthermore, in line with the data from the prison TB surveillance system and prevalence surveys, we assumed that 43% of new infectious cases are detected, that 34% of new non infectious cases are detected and that 65% of treated cases are cured. The transmission rate was calibrated in such a way that the average predicted prevalence of active TB remains roughly stable over this 10 year period. The calibration of the model was done by determining the size of each compartment at time t = 0 and the value of the transmission rate leading to the equilibrium of the deterministic version of the model and fulfilling the constraints on the values of the prevalence of infection and of disease in the prison and at the entry in prison.

Then, we explored the potential effect on active TB prevalence of several simulated strategies (S1 to S5) based on the following control methods, considered alone or associated, as shown in table 3:

- DOTS strategy reaching the WHO target [6]: to detect 70% of new bacteriologically-positive cases and to cure 85% of detected cases
- Systematic detection of TB at entry point of symptomatic (cough>3 weeks) smear-positive cases
- Systematic detection of TB at entry point using chest X-ray
- Annual X-ray mass screening of inmates.

**Table 3:** Description of strategies 1 to 7 and predicted prevalence (%) for the current scenario and for each strategy

	In prison		At entry point		Active TB prevalence (all cases)									Bacteriologically positive TB prevalence								
	DOTS*	Annual systematic X-ray screening	Detection of smear+symptomatic cases	Systematic detection with chest X-ray	Year 3			Year 5			Year 10			Year 3			Year 5			Year 10		
					Median (%)	Mean (%)	P5, P95‡ (%)	Median (%)	Mean (%)	P5, P95 (%)	Median (%)	Mean (%)	P5, P95 (%)	Median (%)	Mean (%)	P5, P95‡ (%)	Median (%)	Mean (%)	P5, P95 (%)	Median (%)	Mean (%)	P5, P95 (%)
**C^+^**	x				4.6	4.6	3.4, 5.9	4.4	4.5	3.1, 6.0	4.3	4.4	2.6, 6.2	3.0	3.0	1.9, 4.1	2.8	2.9	1.7, 4.1	2.8	2.8	1.5, 4.2
**S1**	x				3.3	3.4	2.4, 4.5	2.8	2.8	1.8, 4.0	2.2	2.2	1.3, 3.3	1.7	1.7	1.1, 2.6	1.4	1.4	0.7, 2.1	1.1	1.1	0.4, 1.8
**S2**	x		x		3.3	3.3	2.3, 4.4	2.8	2.8	1.8, 4.0	2.0	2.1	1.1, 3.2	1.7	1.7	0.9, 2.5	1.3	1.3	0.6, 2.2	0.9	1	0.4, 1.7
**S3**	x			x	2.8	2.8	1.9, 3.9	2.1	2.2	1.3, 3.2	1.3	1.4	0.6, 2.4	1.4	1.4	0.8, 2.1	1.1	1.1	0.5, 1.8	0.6	0.7	0.2, 1.3
**S4**	x	x	x		0.8	0.7	0.3, 1.2	0.6	0.6	0.2, 1.1	0.5	0.5	0.2, 1.0	0.4	0.4	0.1, 0.7	0.3	0.3	0.0, 0.6	0.3	0.3	0.0, 0.6
**S5**	x	x		x	0.4	0.5	0.2, 0.8	0.3	0.3	0.0, 0.6	0.2	0.3	0.0, 0.6	0.2	0.2	0.0, 0.5	0.1	0.2	0.0, 0.4	0.1	0.1	0.0, 0.4
**S6**	x	3 screenings†		x	0.5	0.5	0.1, 0.8	0.7	0.7	0.2, 1.2	0.8	0.9	0.3, 1.6	0.2	0.3	0.0, 0.6	0.3	0.3	0.0, 0.7	0.4	0.4	0.1, 0.9
**S7**	x	3 screenings†		2 1^st^ years	0.7	0.7	0.3, 1.2	1.2	1.2	0.6, 1.9	1.7	1.7	0.8, 2.7	0.4	0.4	0.1, 0.7	0.6	0.6	0.2, 1.1	0.8	0.8	0.3, 1.4

+ C = Current scenario

* DOTS: Case detection through quality assured-bacteriology and standardized treatment, with supervision and patient support.

For the current scenario: Passive detection of 43% of new infectious cases, 34% of new non-infectious cases and cure rate of 65% of detected cases

For S1–S7: Passive detection of 70% of new bacteriologically-positive cases and cure rate of 85% of detected cases

x This method is implemented for this strategy

† Screenings at the start and after 1 and 2 years

‡ Percentiles 5 and 95

Considering the cost and the operational complexity of annual X-ray mass screening included in strategies 4 and 5 described in table 3, we simulated strategies (S6 and S7) including a first 2 years phase of the DOTS associated with X-ray detection at entry and annual X-ray mass screening (3 X-ray mass screenings) followed by a second phase limited to DOTS plus X-ray detection at entry (S6) or to DOTS alone (S7).

To implement the DOTS strategy, we modified the values of the proportions of new cases detected (f_1_, f_2_) and the value of the proportion of detected cases cured by treatment (g) as shown in table 1. We considered that smear-negative/culture-negative cases are not detected.

To simulate the systematic detection of TB at entry point (based on symptoms or chest X-ray), we considered that detected cases would be treated as soon as they are detected and we modified the relative proportion of cases entering compartments T, D, Y and R accordingly. X-ray screenings were simulated by moving cases from compartments T, D, Y to compartments Y and R. We considered that X-ray screening allows the detection of 100% of cases, that all detected cases are treated and that the screening of smear-positive symptomatic cases at entry point allows the detection of 21% of bacteriologically positive cases [4]. Other parameter values are presented in table 1.

For the current scenario and for each simulated strategy, we performed 600 runs of the model and computed the mean and the percentiles 5 and 95 of the active TB prevalence at different dates.

### Multivariate uncertainty and sensitivity analyses

Then, to understand how uncertainty on parameters would affect uncertainty on the results obtained for each strategy described in table 3, we performed multivariate uncertainty and sensitivity analyses including all parameters except those defining each strategy (detection and cure rates). We carried out one analysis for the current scenario and one for each of the strategies S1-S7. Using the Latin Hypercube Sampling method (LHS), we generated a sample of set of parameters using distributions of parameter described in table 1 [20], [24]. The active TB average prevalence on 600 runs was considered as the model output. Hence, for each analysis, we computed the average predicted prevalence of active TB after 10 years with each set of parameters of the Latin Hypercube Sample. The same sample was used for the eight analyses.

Then, in order to quantify the impact of the variation of each parameter on the output of the model, we computed the partial rank correlation coefficients (PRCC) between each parameter and the average predicted prevalence of active TB with each strategy [24].

## Results

### Simulations with each strategy

**Figure 2:**
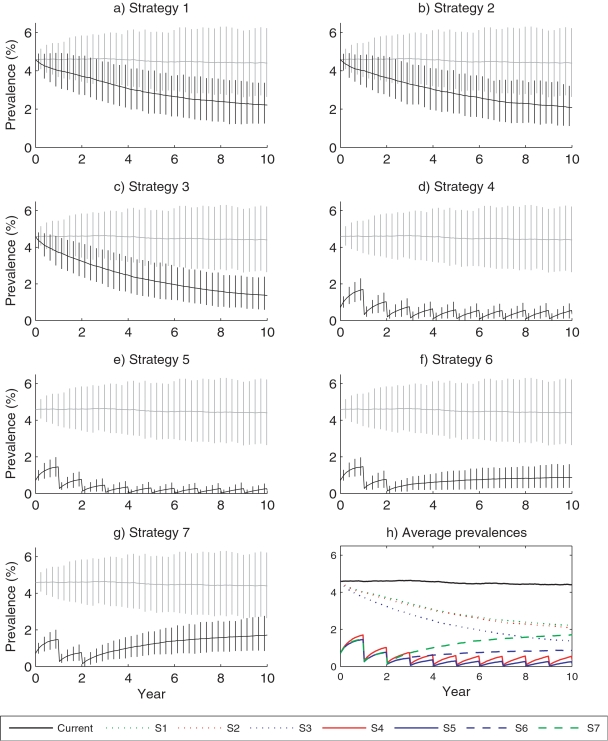
Predicted prevalence (%) of active TB. Simulations are performed over a 10 years period for the seven strategies (black lines) and for the current scenario (grey lines). The continuous line represents the mean of the prevalence on 600 simulations and the vertical line extremities represent percentiles 5 and 95.

Active TB prevalence rates (mean and percentiles 5 and 95) predicted over a 10 year period under the ongoing scenario and under the different strategies (S1–S7) are shown in table 3 and in figure 2.

Assuming that the active TB prevalence would remain stable if the ongoing scenario was applied, we calibrated the transmission rate at 11.10^−3^. With this scenario, the average number of new cases detected during the first year was 1290 [(P5, P95) = (780, 1860)] per 100 000.

When we simulated the implementation of the DOTS strategy meeting the WHO target (S1), the model predicted a slow decreasing trend of active TB average prevalence from 4.6 to 3.4% (2.4, 4.5) at year 3 and 2.2% (1.3, 3.3) at year 10. Adding to this strategy a systematic TB detection at entry point based on symptoms (S2) had no additional effect. Results were slightly improved when mass screening at entry point was based on X-ray (S3).

Considering the limited and slow decreasing trends observed with S1, S2 and S3 strategies, we simulated strategies associating annual mass X-ray plus DOTS and screening at entry point (S4 and S5). When simulating these strategies, we observed a rapid decrease in active TB average prevalence from 4.6 to 0.7% (0.3, 1.2) at year 3 when the screening at entry point was based on symptoms (S4). Active TB average prevalence was slightly lower when the screening at entry point was based on X-ray (S5). In both instances, at year 10, the active TB average prevalence did not exceed 0.5% and the active TB prevalence exceeded 1% in less than 5% of the 600 runs.

We also simulated strategy 5 during 2 years and, then, limited the intervention to DOTS plus X-ray screening at entry point (S6). After the rapid reduction in active TB prevalence mentioned above, the average predicted active TB prevalence remained below 1% until the 10th year.

When we simulated the same strategy (S6) without screening at entry point after the 2 first years (S7), the average active TB prevalence increased faster to reach 1.7% (0.8, 2.7) at year 10. With this last scenario, more than 90% of the simulated active TB prevalences exceeded 1%.

### Multivariate uncertainty and sensitivity analyses

**Figure 3:**
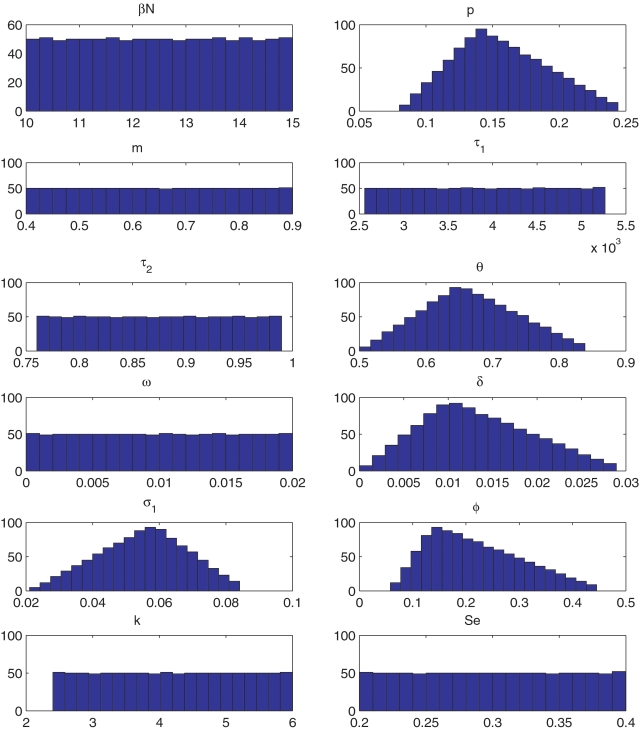
Histograms of the twelve parameters in the sample generated with LHS method.

**Figure 4:**
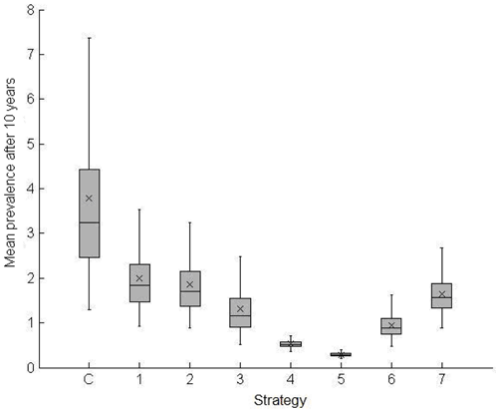
Results of the uncertainty analysis. Boxplots of the average prevalence after 10 years (%). For each set of parameters of the sample generated by the Latin Hypercube Sampling method and each strategy, we performed 600 runs of the model and computed the average prevalence after 10 years. Each boxplot represents the median, the first and third quartiles (Q1 and Q3), the mean and the maximum and minimum values which are in the range [Q1−1.5 IQR, Q3+1.5 IQR] with IQR equal to the inter-quartile range (Q3-Q1).

The empiric distributions of parameters in the LHS are represented in figure 3. According to our sensitivity analysis, the uncertainty of our predictions was much lower with S4, S5 and S6 than with other strategies (see figure 4). Indeed, with S1, S2, S3 and S7, the average predicted prevalence of active TB after 10 years could reach 2.5% or more for several of the sets of parameters generated with the LHS method, whereas the average predicted prevalence of active TB with S4, S5 and S6 after 10 years was below 0.7, 0.4 and 1.5% respectively for 95% of these sets of parameters.

The sensitivity analysis showed that, for all strategies, the parameters whose variations had the greatest impact on our predictions at 10 years were the proportion of fast progressors, the death rate of untreated TB, the partial immunity afforded by previous infection, the transmission rate and the rate at which detected cases would be detected (see table 4).

**Table 4:** Partial rank correlation coefficient (PRCC) between each parameter and the average predicted prevalence after 10 years, for the current scenario and TB control strategies S1–S7.

Definition parameter		Partial rank correlation coefficient							
		C	S1	S2	S3	S4	S5	S6	S7
β	Transmission rate	**0. 75**	**0. 75**	**0. 74**	**0. 77**	**0. 67**	**0. 62**	**0. 76**	**0. 76**
p	Proportion of fast progressors	**0. 93**	**0. 92**	**0. 92**	**0. 93**	**0. 85**	**0. 82**	**0. 92**	**0. 92**
m	Partial immunity afforded by previous infection	**−0. 91**	**−0. 85**	**−0. 85**	**−0. 85**	**−0. 63**	−0. 57	**−0. 79**	**−0. 81**
τ_1_	Rate at which slow progressors develop TB	0. 31	0. 48	0. 49	0. 58	**0. 85**	**0. 92**	**0. 76**	**0. 61**
τ_2_	Rate at which fast progressors develop TB	0. 25	0. 22	0. 21	0. 22	0. 22	0. 27	0. 26	0. 28
θ	Proportion of TB cases who become infectious	0. 59	0. 18	0. 10	0. 33	−0. 55	0. 04	0. 32	0. 14
ω	Rate of smear conversion	0. 09	0. 15	0. 14	0. 15	0. 04	0. 07	0. 13	0. 12
δ	Rate of relapse	0. 17	0. 26	0. 28	0. 36	0. 58	**0. 72**	0. 52	0. 35
σ_1_	Rate of self cure for non treated infectious cases	−0. 34	−0. 41	−0. 40	−0. 39	−0. 16	−0. 11	−0. 34	−0. 39
ϕ	Death rate of untreated TB	**−0. 91**	**−0. 94**	**−0. 94**	**−0. 93**	**−0. 68**	−0. 52	**−0. 91**	**−0. 93**
k	Rate at which detected cases are detected and treated	−0. 31	**−0. 62**	−0. 59	−0. 57	**−0. 86**	**−0. 79**	**−0. 66**	**−0. 70**
Se	Sensitivity of the detection of smear+symptomatic cases at entry point	0. 02	0. 02	−0. 17	0. 02	−0. 46	0. 00	0. 03	0. 02

PRCC over 0.6 in absolute values are in bold.

The rate at which slow progressors develop TB had also an important impact on the predicted prevalence of active TB after 10 years for strategies including an annual mass screening during ten years (S4 and S5) and, in a less marked way, for strategies including a mass screening limited to 2 years (S6 and S7). Furthermore, for S5, the average predicted prevalence of active TB was impacted by the rate of relapse.

For all strategies, the transmission rate, the proportion of fast progressors and the rate at which slow progressors develop active TB were positively correlated with the average predicted prevalence of active TB; thus overestimating one of these parameters would lead to underestimate the efficacy of the simulated strategies. On the contrary, for all simulated strategies, the level of partial immunity afforded by previous infection, the death rate of untreated TB and the rate at which TB cases would be detected and treated were negatively correlated with the average predicted prevalence of active TB.

## Discussion

According to the predictions of our model applied to a prison with features similar to other prisons in RJ, the association of the DOTS strategy with annual X-ray mass screenings would allow to obtain a rapid and sustained decline in active TB prevalence. Furthermore, after reducing the TB burden by implementing three annual X-ray mass screenings, the sole association of X-ray screening at entry point and DOTS could be sufficient to maintain a low active TB prevalence level during several years. The better performance of strategies including annual X-ray screening can be explained by the assumption that X-ray screenings allow the rapid diagnosis of all active TB cases, including asymptomatic and not yet infectious cases which are not detected by the DOTS strategy. By this way, the pool of active TB cases decreases drastically although it is then increased by the development of TB among infected individuals, treatment failure, relapse from treatment and by active TB cases entering the prison for strategies which do not include X-ray screening at entry. The limited performance of DOTS on its own in this highly endemic setting can be explained by the fact that it does not decrease rapidly the pool of active TB cases.

We have shown that the uncertainty of our predictions was less important for strategies which included an annual mass screening (S4, S5). For these strategies, the rate at which slow progressors develop TB was one of the parameters whose variations had the greatest impact on the predicted active TB prevalences. Indeed, these strategies would decrease rapidly the prevalence of infectious cases in the prison; as a consequence, the role of exogenous infections in TB transmission would be reduced, and the relative contribution of endogenous reactivation to the incidence of active TB would be larger.

The value of the transmission rate had also an important impact on our predictions. It was calibrated so that, with the current scenario, the active TB prevalence would remain roughly stable over 10 years. The average incidence we predicted was in line with the annual incidences observed during the years prior to our prevalence survey in the prison we investigated [2]. This value of the transmission rate is in the high range of values used in previously published models concerning general populations [12], [25], a finding consistent with results of previous studies [26] showing the strong relationship of overcrowding with TB transmission in a highly endemic prison. However, for various reasons including a greater transmission rate or a larger proportion of recent infections, the active TB prevalence may increase rather than being stable in some prisons. Under this circumstance, all strategies may be less effective. Nonetheless, the performance of strategies including X-ray screening should remain more effective as all active TB cases would be detected each year.

In our multivariate sensitivity analysis, we did not include the sensitivity of X-ray as a screening method, considering it was 100% in agreement with results of previous studies which evaluated at 0.97 (0.90; 1.0) the sensitivity of any chest X-ray abnormality for detecting bacteriologically positive TB cases [22]. If the sensitivity was smaller, our predictions could have overestimated the relative impact of strategies including X-ray screening. Furthermore, in the present study, we limited our simulations of mass screening to X-ray based screening. Indeed, mass screening based on symptoms, commonly used in prevalence surveys [8], [27], [28] and recommended in prison's TB control program [7], performed poorly when compared with X-ray based screening [4], [22], [29]–[31].

The need for longitudinal studies concerning the impact of TB screening at entry point has been recently underlined [11]. Using our model, the comparison of predicted active TB prevalence trends observed under strategy 7 versus strategy 6 shows that, in the highly endemic prison we investigated, X-ray screening at entry point would have a greater impact when active TB prevalence is reduced after three annual X-Ray mass screenings. In the case of high active TB prevalence, given the heavy circulation of Mycobacterium tuberculosis (MTB) within the prison, the contribution of infectious TB cases among inmates at entry to this overall MTB circulation is relatively limited, even if the active TB prevalence at entry is high as observed in the prison we studied (1.5%). In our study, we considered that the prevalence of TB infection and active TB among inmates at entry point is constant during the whole ten year study period. The high active TB prevalence at entry point could be decreased in RJ state prisons by reducing the major overcrowding and introducing health care in the police remand where the convicts are incarcerated for periods of time which can last for more than 6 months before they are transferred to prison units. The effect of this strategy could be evaluated by using our model.

Aimed at providing evidences to guide decision makers, the model we propose concerning a Brazilian prison can be applied to other overcrowded institutions with different level of TB prevalence at entry point and inside the institution. Available incidence and prevalence data suggest that a similar TB situation prevails in most RJ state prisons [2], [5] where our strategic conclusions may apply as well as to other highly endemic prisons and institutions worldwide. However, we must keep in mind that an underlying assumption of our model is the homogeneous mixing of the population investigated. Due to overcrowding conditions in collective cells, this assumption is probably appropriate for the prison we studied, but may not be valid for units where the number of inmates per cell and the circulation of inmates are limited, such as high security units.

Given the relatively low level of HIV seroprevalence in our study population (2.1%), we did not take into account in our model the effect of HIV-infection on TB [4]. In many prisons worldwide, the HIV seroprevalence is much higher [32], [33] and HIV infection should be considered given its interactions with TB [34]. Further, the drug resistance was not a major problem in the prison we studied [4] and in other prisons we subsequently studied in RJ State [5]. Therefore, we did not consider this issue and our model should be modified on the basis of previously published studies [13], [14], [17]–[19] when applied to prisons where drug resistance is a major problem.

The predictions based on our model show that the impact of the DOTS strategy alone, even if it reaches the WHO objectives of 70% of bacteriologically positive cases detected and 85% of detected cases cured, is too slow to face the urgent situation of high TB endemicity in the prison we investigated and in similar settings. Indeed, the DOTS strategy should remain the basic tool to control TB in prison but, given its limited impact in the case of high active TB prevalence, active detection, preferentially based on X-ray, should be considered at entry in prison and among inmates already incarcerated.

Several surveys performed in RJ prisons [4], [5] and elsewhere [30] demonstrated the feasibility of X-ray screening and its excellent acceptance by inmates. Finally, our model may provide the elements for cost-effectiveness analysis of tuberculosis control approaches which need to be explored in further research.
